# An Unbiased Genetic Screen Reveals the Polygenic Nature of the Influenza Virus Anti-Interferon Response

**DOI:** 10.1128/JVI.00014-14

**Published:** 2014-05

**Authors:** Maite Pérez-Cidoncha, Marian J. Killip, Juan C. Oliveros, Víctor J. Asensio, Yolanda Fernández, José A. Bengoechea, Richard E. Randall, Juan Ortín

**Affiliations:** a Department of Molecular and Cellular Biology, Centro Nacional de Biotecnología (CSIC), Madrid, Spain; b School of Biology, Centre for Biomolecular Sciences, University of St Andrews, St Andrews, Fife, United Kingdom; c Fundació d'Investigació Sanitària de les Illes Balears, Recinto Hospital Joan March, Bunyola, Spain; d Laboratory Microbial Pathogenesis, Fundació d'Investigació Sanitària de les Illes Balears, Recinto Hospital Joan March, Bunyola, Spain; e Ciber de Enfermedades Respiratorias, ISCIII, Mallorca, Illes Balears, Spain

## Abstract

Influenza A viruses counteract the cellular innate immune response at several steps, including blocking RIG I-dependent activation of interferon (IFN) transcription, interferon (IFN)-dependent upregulation of IFN-stimulated genes (ISGs), and the activity of various ISG products; the multifunctional NS1 protein is responsible for most of these activities. To determine the importance of other viral genes in the interplay between the virus and the host IFN response, we characterized populations and selected mutants of wild-type viruses selected by passage through non-IFN-responsive cells. We reasoned that, by allowing replication to occur in the absence of the selection pressure exerted by IFN, the virus could mutate at positions that would normally be restricted and could thus find new optimal sequence solutions. Deep sequencing of selected virus populations and individual virus mutants indicated that nonsynonymous mutations occurred at many phylogenetically conserved positions in nearly all virus genes. Most individual mutants selected for further characterization induced IFN and ISGs and were unable to counteract the effects of exogenous IFN, yet only one contained a mutation in NS1. The relevance of these mutations for the virus phenotype was verified by reverse genetics. Of note, several virus mutants expressing intact NS1 proteins exhibited alterations in the M1/M2 proteins and accumulated large amounts of deleted genomic RNAs but nonetheless replicated to high titers. This suggests that the overproduction of IFN inducers by these viruses can override NS1-mediated IFN modulation. Altogether, the results suggest that influenza viruses replicating in IFN-competent cells have tuned their complete genomes to evade the cellular innate immune system and that serial replication in non-IFN-responsive cells allows the virus to relax from these constraints and find a new genome consensus within its sequence space.

**IMPORTANCE** In natural virus infections, the production of interferons leads to an antiviral state in cells that effectively limits virus replication. The interferon response places considerable selection pressure on viruses, and they have evolved a variety of ways to evade it. Although the influenza virus NS1 protein is a powerful interferon antagonist, the contributions of other viral genes to interferon evasion have not been well characterized. Here, we examined the effects of alleviating the selection pressure exerted by interferon by serially passaging influenza viruses in cells unable to respond to interferon. Viruses that grew to high titers had mutations at many normally conserved positions in nearly all genes and were not restricted to the NS1 gene. Our results demonstrate that influenza viruses have fine-tuned their entire genomes to evade the interferon response, and by removing interferon-mediated constraints, viruses can mutate at genome positions normally restricted by the interferon response.

## INTRODUCTION

The influenza A viruses are members of the family Orthomyxoviridae and cause annual epidemics and occasional pandemics of respiratory disease with considerable public health and economic impact (1, 2). The viral genome comprises eight single-stranded RNA molecules of negative polarity, which are encapsidated into distinct ribonucleoprotein particles (RNPs) containing the viral polymerase and multiple nucleoprotein (NP) monomers (3–7). Upon recognition of sialic acid-containing cellular receptors, the virus enters the cell by endocytosis and the viral genome reaches the cytoplasm by hemagglutinin-mediated membrane fusion at the late endosome (8). Transcription and replication of the viral RNPs occur in the nucleus and are mediated by the polymerase, a heterotrimer composed of the PB1, PB2, and PA subunits. For transcription, the polymerase uses a cap-snatching mechanism to generate viral mRNAs, whereas replication leads to the production of progeny RNPs similar to those present in the virion (3, 4, 6). Finally, progeny RNPs are exported from the nucleus by a CRM1-dependent pathway and become incorporated in a specific manner into virus particles. These progeny virions bud from lipid rafts at the plasma membrane in a process that requires the specific action of the M1 and M2 proteins (9–13).

As a consequence of influenza infection, the cell mounts an innate immune response to restrict virus multiplication. The main driver of this response is a group of cytokines collectively named type I interferons (IFNs) (14). Expression of IFNs requires the detection of virus-specific patterns (pathogen-associated molecular patterns [PAMPs]), which are recognized as nonself by cellular sensors (pattern recognition receptors [PRRs]) (15), of which RIG-I is relevant during the infection of epithelial cells by influenza virus (16–18). RIG-I becomes activated by recognition of double-stranded RNA (dsRNA) containing a 5′-triphosphate (19, 20) and signals downstream through an interaction with the mitochondrial protein IPS-1/MAVS/VISA/Cardif. The consequence of this downstream signaling is the activation of the transcription factors IRF3, NF-κB, and ATF-2/c-Jun, which activate IFN transcription (reviewed in reference 21). Secreted IFNs act in an autocrine and paracrine fashion by activating the IFN receptor. The signal is transmitted by the Jak/STAT pathway to the nucleus, where it activates the transcription of hundreds of IFN-dependent genes (ISGs), many of which have antiviral activity (PKR, Mx, OAS, etc.) to help the cell to overcome virus infection and restrict virus spread to neighboring uninfected cells (14, 22).

Viruses have evolved a variety of countermeasures to avoid cellular innate immunity and maintain reasonable replication efficiency. Some viruses can block the activation of PAMP sensors or inhibit downstream signaling, thereby diminishing IFN expression. Other viruses counteract IFN activity by targeting various stages of signaling downstream from IFN receptor, and, finally, many viruses directly inhibit the activity of one or several antiviral ISG products (reviewed in reference 14). In addition, many lytic viruses diminish the level of transcription and/or translation of all cellular genes and hence indirectly downregulate the induction of IFNs and ISGs. In the case of influenza A viruses, the nonstructural NS1 protein is the main IFN-counteracting factor. NS1 is a small multifunctional protein involved in viral protein synthesis, viral RNA replication, and virion production (23–27) that also modulates cellular posttranscriptional RNA processing and transport (28–31) (reviewed in reference 32). Influenza A viruses lacking NS1 protein expression cannot multiply in normal, IFN-competent cells but are viable in cellular systems deficient in innate immunity, although viral yields are still reduced compared to those of wild-type virus (33, 34). This indicates that the primary but not the only role of NS1 is to counteract the cellular IFN response. This is achieved through a combination of several NS1-host cell interactions, some of which are strain specific (35). These include (i) inhibiting cellular transcription elongation and posttranscriptional RNA processing (28, 36, 37), (ii) blocking RIG-I activation (16–18, 38), (iii) interfering with IFN signaling (39, 40), and (iv) directly inhibiting specific ISG products, such as PKR and RNase L (41–43).

In addition to NS1, other influenza virus genes have been reported to alter the cellular IFN response; the viral polymerase inhibits activation of IPS-1/MAVS, possibly as a consequence of PB2 localization at the mitochondria (44, 45), while PB1-F2 protein appears to modulate the IFN response, although contradictory results have been reported (46–49). In this report, we have undertaken a nonbiased genetic approach to determine the viral genes that normally play a role in modulating the cellular IFN response. Our results show that the mutations that appear in virus populations upon serial passage in non-IFN-responsive cells not only are detected in the main IFN response modulator NS1 but also occur in essentially every viral gene. Moreover, most of the virus mutants identified and subsequently characterized induce more IFN than wild-type (wt) virus but do not contain alterations in NS1. Interestingly, several of these mutants are particularly prone to accumulating deletion-containing RNAs derived from the genes encoding the polymerase subunits.

## MATERIALS AND METHODS

### Biological materials.

In the course of this study, the following influenza A strains were used: A/Victoria/3/75 (VIC), A/Puerto Rico/34 (PR8), and ΔNS1 (33). Encephalomyocarditis virus (EMCV) was used for the IFN bioassay. The MDCK cell line was purchased from the ATCC. The A549 cell line (50) was obtained from J. A. Melero. The origin of MDCK-V2 and MDCK-V5 cell lines has been described (51), as well as the generation of A549/pr(IFN-β).GFP cells (52, 53). A549 cells stably expressing luciferase under an interferon-stimulated response element (ISRE) promoter [A549/pr(ISRE).Luc] were a kind gift from G. Adolf, Boehringer Ingelheim, Austria, and were engineered to express BVDV/NPro [A549/pr(ISRE).Luc-BVDV-N^pro^] to render them IRF3 deficient and unable to generate IFN (54).

Antibodies used in these procedures included monoclonal antibodies against β-actin (Sigma), phospho-Akt (Ser473; Cell Signaling Technology), and polyclonal antibodies to ISG56 (Santa Cruz), MxA (Santa Cruz), phospho-IRF3 (Ser396; Cell Signaling Technology), Akt (pan; Cell Signaling Technology), cleaved caspase-3 (Asp175; Cell Signaling Technology), phospho-eIF2α (Ser51; Cell Signaling Technology), and STAT1 (Cell Signaling Technology). The specific anti-NS1 and anti-NP rabbit antibodies (55, 56), anti-M1 mouse antibodies (12), and anti-NS1 rat antibodies (24) were described previously.

### Virological techniques.

The influenza virus plaque assay was carried out on MDCK-V2 cells as described previously (57). Viral plaques were stained with crystal violet or by immunocytochemistry using sheep antisera raised against purified and disrupted X31 (H3N2) virus (anti-X31; Diagnostics Scotland) (58). For serial passage, a virus stock generated by three consecutive steps of limiting dilution was used as initial inoculum. Infections were carried out in sextuplicate at a multiplicity of infection of 10^−3^ PFU per cell on either MDCK or MDCK-V2 cultures (10^7^ cells). When complete cytopathic effect was observed, the supernatant was collected, clarified, and used for a further infection cycle. The titers of each serial passage virus ranged between 5 × 10^7^ and 2 × 10^8^ PFU per cell. For virus purification, virus supernatants were clarified by centrifugation for 10 min (10,000 × *g*, 4°C) and sedimented through a 33% sucrose cushion in TNE (50 mM Tris HCl–100 mM NaCl–10 mM EDTA, pH 7.5) for 1 h (28,000 × *g*, 4°C). The virus pellet was resuspended in TNE and sedimented again on a 50-to-33% sucrose step gradient in TNE. The virus-containing interface was collected, diluted in TNE, and sedimented under the same conditions (24).

IFN activity in the culture supernatants was determined by a cytopathic effect (CPE) reduction bioassay. Culture supernatants from cells infected at 5 PFU/cell were harvested after 24 h postinfection (hpi) and centrifuged at 1,500 × *g* for 10 min to eliminate cellular debris. After UV treatment to inactivate residual virus, the supernatants was serially diluted 2-fold and added to A549/BVDV-Npro cell monolayers for 24 h prior to infection with EMCV at 0.05 PFU/cell. Monolayers were fixed after 2 to 3 days and stained with crystal violet to monitor CPE. The number of wells protected were converted to IFN bioassay arbitrary units using an IFN-α standard.

### Mutant virus screening.

For cell sorting, plates of A549/pr(IFN-β).GFP cells were infected at 0.04 FPU/cell. At 8 hpi, cells were trypsinized, resuspended in Mg^2+^- and Ca^2+^-free phosphate-buffered saline (PBS), passed through a 0.4-mm-pore-size filter to obtain single-cell suspensions, and sorted in an Influx cell sorter (BD Bioscience). Fluorescence-activated cell sorting (FACS) analysis was performed on A549/pr(IFN-β).GFP cells following trypsinization to obtain single-cell suspensions and fixation in PBS–1% formaldehyde. Expression of green fluorescent protein (GFP) was examined using a BD FACScan flow cytometer, and data were analyzed using FlowJo (Treestar).

Specific mutations were engineered into recombinant virus genomic pHH plasmids derived from the VIC strain using the QuikChange site-directed mutagenesis kit from Stratagene as recommended by the manufacturer. The mutations were rescued into infectious virus by standard techniques (24, 59).

### Protein analyses.

Western blotting and immunofluorescence were carried out as described previously (60, 61). For immunofluorescence, infected A549 cells were washed with PBS, fixed with 1% paraformaldehyde, and permeabilized with 0.5% Triton X-100 for 5 min. The cells were blocked with PBS–3% bovine serum albumin (BSA and incubated with primary antibody diluted in PBS–1% BSA for 1 h at room temperature. After washing with PBS, coverslips were further incubated with goat anti-rabbit or goat anti-rat antibodies bound to Alexa 488 or Alexa 594 fluorochromes. Coverslips were mounted with Prolong reagent and analyzed by confocal microscopy using a Leica TSC SP5 microscope.

For the luciferase assay, A549/pr(ISRE).Luc or A549/pr(ISRE).Luc-BVDV-N^pro^ cells were infected at 5 FPU/cell and were treated 7 h later with IFN-α (Roferon A; Roche) at 10^4^ units/ml or left untreated. At 13 h postinfection, luciferase expression was determined using a luciferase assay system (Promega).

### RNA analyses.

The RNA present in purified virions was purified by treatment with 0.5% SDS and 200 μg/ml proteinase K in TNE for 30 min at 37°C followed by extraction with phenol-chloroform-isoamyl alcohol-hydroxyquinoline and ethanol precipitation (62). Purified RNA was separated by electrophoresis on denaturing 4% polyacrylamide–6 M urea gels and silver stained as described previously (62). Reverse transcription-PCR (RT-PCR) for PB1, PB2, and PA segments was performed to determine the presence of deletion-containing RNAs. To detect the different segments, the oligonucleotide 5′-GTCACGTCTCATATTAGTAGAAACAAGG-3′ was used in combination with either 5′-AGCAAAAGCAGGTCAATTATATTCAATATG-3′ to detect PB2, 5′-CAAAAGCAGGCAAACCATTTGAA-3′ to detect PB1, or 5′-GCAAAAGCAGGTACTGATTCGAGA-3′ to detect PA (virus-specific sequences are underlined). The reverse transcription reaction was performed for 30 min at 42°C, and then PCR was performed for 30 rounds of 94°C for 30 s, 55°C for 30 s, and 68°C for 2.5 min with a final extension time of 7 min at 68°C.

### Deep sequencing.

Deep sequencing of virus populations was performed using the Illumina HiSeq2000 sequencer with the sequencing kit, version 5. After removal of the four nucleotide bar codes used for multiplexing, more than 4 million single-end 76-nucleotide (nt) reads were obtained for each sample. Deep sequencing of selected virus mutants was performed with the Illumina HiSeq2000 sequencer using the HiSeq sequencing kit TruSeq v3. In these cases, more than 20 million paired-end 46-nt reads were obtained.

### Sequence analysis.

For alignment of short reads against the viral genome, the BWA algorithm (63) was employed, using the sequence of VIC as a reference. In all cases BWA alignment parameters were established to allow 5 mismatches and no gaps (*n*, 5; *o*, 0). SAMtools (64) were used for the management of the files generated by BWA. The following strategy was used for detecting genomic deletions. Every 46-nt read was split into two 23-nt fragments. Each fragment was then independently aligned against the VIC genome with BWA software, allowing up to 1 mismatch and no gaps. The alignments of two fragments of a single 46-nt read in the same strand of the same viral segment at a distance 38 or more nucleotides (indicating a gap of 15 or more nucleotides) were scored as positive and retrieved. To obtain other junction-compatible read alignments that do not lie in the exact middle of a 46-nt read, the entire process was repeated with fragments of 20 to 26 nt, 21 to 25 nt, 22 to 24 nt, 24 to 22 nt, 25 to 21 nt, and 26 to 20 nt. For each sample, all junction-compatible alignment events with a gap size of 15 nt or more and that occurred in at least 2 different reads were considered for subsequent analysis.

## RESULTS

**FIG 1:**
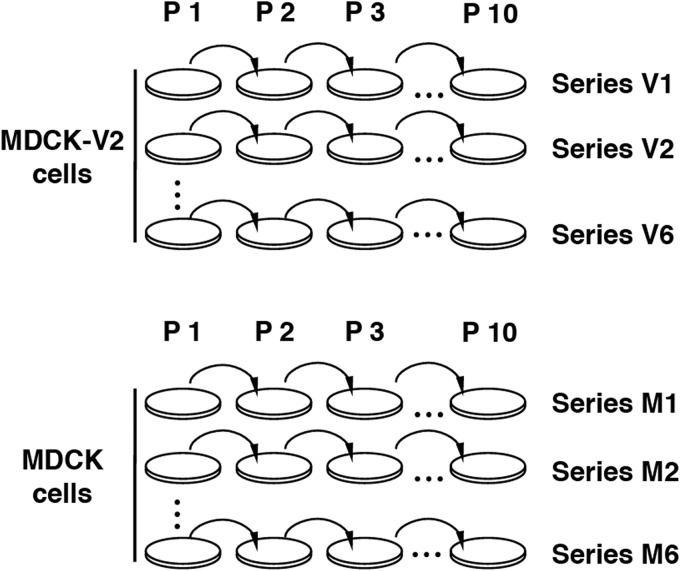
Diagram of virus serial passage history. MDCK-V2 or MDCK cell cultures were serially infected at a multiplicity of infection around 0.001 PFU/cell with A/Victoria/3/75 virus. The initial infecting virus was generated by three steps of endpoint dilution to limit the presence of defective virus particles. Six replicates were carried out in each cell line, generating passage series V1 to V6 (in MDCK-V2 cells) and M1 to M6 (in MDCK cells).

Most of the experimental evidence regarding how influenza virus circumvents the IFN response derives from studies of the phenotype of NS1 deletion or point mutants and from protein-protein interaction studies involving NS1. In contrast, here we performed an unbiased genetic screen to identify the viral genes that influence the outcome of IFN-mediated innate immune responses to influenza virus infections. Instead of increasing the IFN selection pressure (65), we carried out serial passage of A/Victoria/3/75 (VIC) influenza virus in non-IFN-responsive cells to release the virus from sequence constraints imposed by the IFN response. A diagram showing the experimental strategy employed is presented in Fig. 1. A wt influenza virus was passaged serially in either MDCK or MDCK-V2 cells, which express the V protein of parainfluenza virus type 2 (PIV2) and are thus insensitive to IFN-α/β, since V targets STAT1 for degradation in order to block IFN signaling (51). Cells were infected at a low multiplicity of infection (MOI) (0.001 PFU/cell) to (i) select for efficiently replicating viruses and (ii) prevent the accumulation of defective virus (66). Six independent serial passages were carried out in parallel to increase the diversity of potential mutants generated.

### Phenotypic and genetic analyses of passaged virus populations.

**FIG 2:**
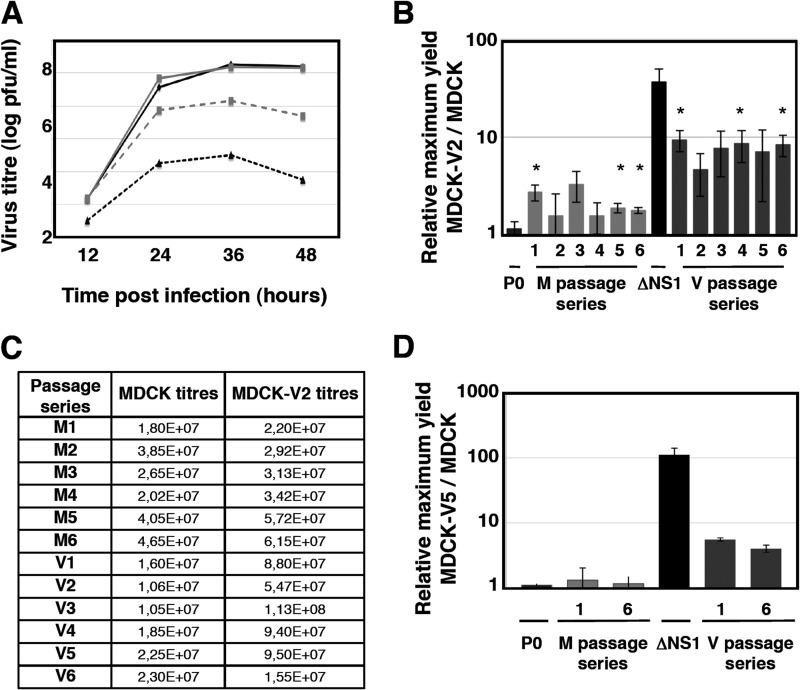
Replication efficiency of serially passaged viruses in MDCK and non-IFN-responsive MDCK cells. (A) Replication kinetics of wt and ΔNS1 viruses in MDCK-V2 (gray lines) and MDCK cells (black lines). Cell cultures were infected with the initial virus stock (solid lines) or ΔNS1 virus (dotted lines) at an MOI of 0.001. At the indicated times after infection, samples were obtained from the culture supernatant, and the viral titers were determined by plaque assay in MDCK-V2 cells. (B) The efficiency of replication of the various viruses of the M and V passage series were evaluated by low-multiplicity infections in MDCK-V2 and MDCK cells after 10 serial passages. Cultures were infected with either the initial virus stock (P0), one of the M1 to M6 or V1 to V6 viruses, or ΔNS1 virus at a multiplicity of 0.001 PFU/cell. At various times after infection, samples were collected and titers were determined on MDCK-V2 cells. Data are ratios of maximal titers of infections in MDCK-V2 to those in MDCK cells, as shown in panel A (averages and standard deviations for 3 replicates). *, *P* < 0.05. (C) Absolute titers of a representative kinetics experiment. (D) The efficiency of replication of some viruses of the M and V passage series was evaluated by using low-multiplicity infections in either MDCK-V5 and MDCK cells after 10 serial passages. Cultures were infected with either the initial virus stock (P0) or the M1, M6, V1, V6, or ΔNS1 virus at a multiplicity of 0.001 PFU/cell. At various times after infection, samples were collected and titers were determined on MDCK-V2 cells. Data are ratios of maximal titers of infections in MDCK-V2 to those in MDCK cells, as shown in panel A (averages and standard deviations for 3 replicates). *, *P* < 0.05.

After 10 serial passages, the relative efficiency of replication of the various passaged stocks was evaluated. MDCK and MDCK-V2 cells were infected at a low MOI (0.001 PFU/cell) with the parental virus, ΔNS1 virus, or the passage 10 virus series M1 to M6 and V1 to V6. At various times after infection, the titer of infectious virus was assayed on MDCK-V2 cells and the ratios of the maximum titers obtained following replication in MDCK-V2 cells to those in MDCK cells were determined. The parental virus used for serial passage produced similar yields in both cell lines, while the ΔNS1 virus replicated to approximately 50-fold-higher titers in MDCK-V2 cells than in MDCK cells (Fig. 2A). Viruses serially passaged in MDCK cells produced about 2-fold-higher titers in MDCK-V2 cells than in MDCK cells, but strikingly, viruses that had been selected by passage through MDCK-V2 cells replicated significantly better in MDCK-V2 cells than MDCK cells (approximately 10-fold) (Fig. 2B and C), strongly suggesting that some of the viruses within this population do indeed replicate better than wt virus in non-IFN-responsive cells but are presumably selected against in IFN-competent cells. To confirm this, some of the passage 10 virus populations of the V and M series were used to infect either MDCK or MDCK-V5 cells, a different cell line that expresses the V protein of parainfluenza virus type 5 (PIV5), which also blocks IFN signaling (51). Similar results were obtained; specifically, the viruses passaged in MDCK-V2 cells yielded higher titers in MDCK-V5 cells than in parental MDCK cells, whereas viruses passaged in MDCK cells did not (Fig. 2D).

The phenotype of the passaged virus populations might be due to the presence of many mutations affecting several genes or to a small number of mutations concentrated in one viral gene, for example, NS1. If the latter scenario was the case, then it would be expected that deep sequencing of the viral populations would reveal a greater level of variation in one particular viral gene than the others. To determine whether this was the case, we carried out deep sequencing of purified RNA from passage 10 virions of the V or M series. Under the conditions used, an average of 2,342 reads per position were obtained, and no overall change in the consensus sequence was observed. Moreover, no specific position could be identified that showed significant sequence variation in the V1-V6 passage series compared to the viruses passaged in MDCK cells (data not shown). These results suggested that many different mutations across the virus genome may account for the phenotype observed.

### Screening and characterization of relevant virus populations.

In view of these sequencing results, we next attempted to isolate individual mutant viruses from within the population of viruses selected by passage through MDCK/V2 cells that activated the IFN induction cascade upon infection. In A549 cells engineered to express GFP under the control of the IFN promoter [A549/pr(IFN-β).GFP cells] (52), GFP expression acts as a marker for activation of the IFN induction pathway. These cells were infected with each of the passage 10 V1-V6 virus populations or the M1 MDCK-passaged virus and sorted for GFP expression (the M1 MDCK-passaged virus could contain spontaneous mutants affected in IFN counteraction but was included to set a background value). This sorting strategy would reveal mutant viruses able to induce high levels of IFN but might not identify mutants unable to counteract IFN signaling. The infections were carried out at low multiplicity (0.04 PFU/cell) to reduce the possibility of dual infection of a single cell. Two sorting strategies were used: 1,000 to 9,000 bulk positive cells from each passaged population were collected and plated onto cultures of MDCK-V2 cells to rescue the selected population of progeny viruses, or alternatively, single positive cells were plated onto MDCK-V2 cells to rescue individual mutant viruses (see below).

The genotypes of the bulk virus populations passaged in MDCK-V2 cells and selected by cell sorting (populations VS1 to VS6) were analyzed by deep sequencing of virion RNA on the Illumina platform, using viruses from one of the populations passaged in MDCK cells and sorted in parallel after infection of A549/pr(IFN-β).GFP cells as a control (population MS). The average sequence variability per genome position in all populations was 0.35%. Therefore, we chose 5% (a >10-fold increase in variability) as the limit to identify potential nucleotide changes in the selected population of viruses that may influence the ability of influenza A virus to circumvent the IFN response. A number of positions in the genome of these selected populations (populations VS1 to VS6) showed a significant increase in sequence variation using this criterion and were not particularly variable in the control selected MS population that was passaged in MDCK cells (Table 1). The 41 positions that were specifically variable in populations VS1 to VS6 mapped to all viral RNA segments except NP and identified a potential landscape of mutations associated with an enhanced ability to activate the IFN response or a diminished ability to inhibit it. Among sequence positions that were specifically variable in populations V1 to V6, 11 showed high variability in more than one passage series (Table 1). This suggests that they mutated independently during passaging and hence may represent positions relevant for the regulation of IFN induction. In addition, 24 of the variable positions showed changes that would lead to 25 amino acid substitutions in viral proteins (Table 1). When the nucleotide changes detected in these 41 variable positions were compared to the influenza virus sequence databases, many of the synonymous changes corresponded to positions that are not phylogenetically conserved (13 out of 17) (Table 1); however, most of the amino acid substitutions were observed at positions that are hightly phylogenetically conserved (18 out of 25) (Table 1). These observations suggest that the many synonymous changes imply random drift, whereas the nonsynonymous changes were positively selected for during passage through MDCK-V2 cells, with some of these mutations resulting in viruses that more readily activate the IFN induction cascade, since they also induced expression of GFP in the A549/pr(IFN-β).GFP cells. As this analysis was done at the population level, it is not possible to know whether some of the synonymous changes were coselected with those involving amino acid alterations.

**TABLE 1:**
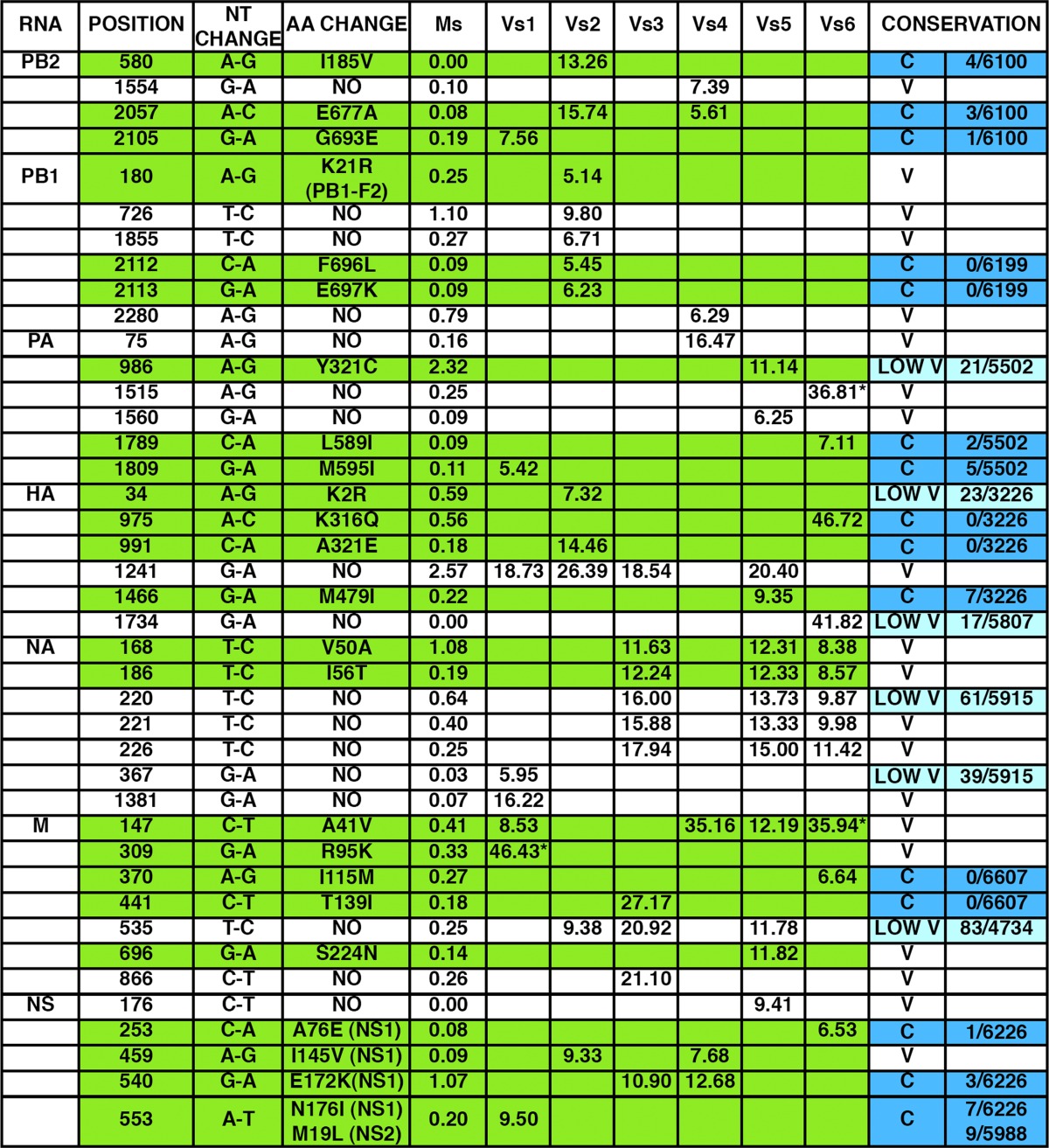
Mutations detected in passaged virus populations^*a*^

a The frequency of specific mutations detected in virus populations passaged in MDCK-V2 cells and sorted for GFP expression (populations VS1 to VS6) is presented, using 5% as the lower threshold. For comparison, the frequency of these mutations in a control population passaged in MDCK cells and sorted for GFP expression (MS) is also presented. The conservation of these positions in the influenza sequence database is denoted as conserved (C; blue), low variable (LOW V; light blue; 10 < *n* < 100), or variable (V; *n* > 100), and the number of instances each mutation appears among the total number of sequences screened is indicated. The mutations leading to amino acid change are highlighted in green, and the amino acid change is indicated.

### Identification and phenotypic characterization of individual virus mutants.

**FIG 3:**
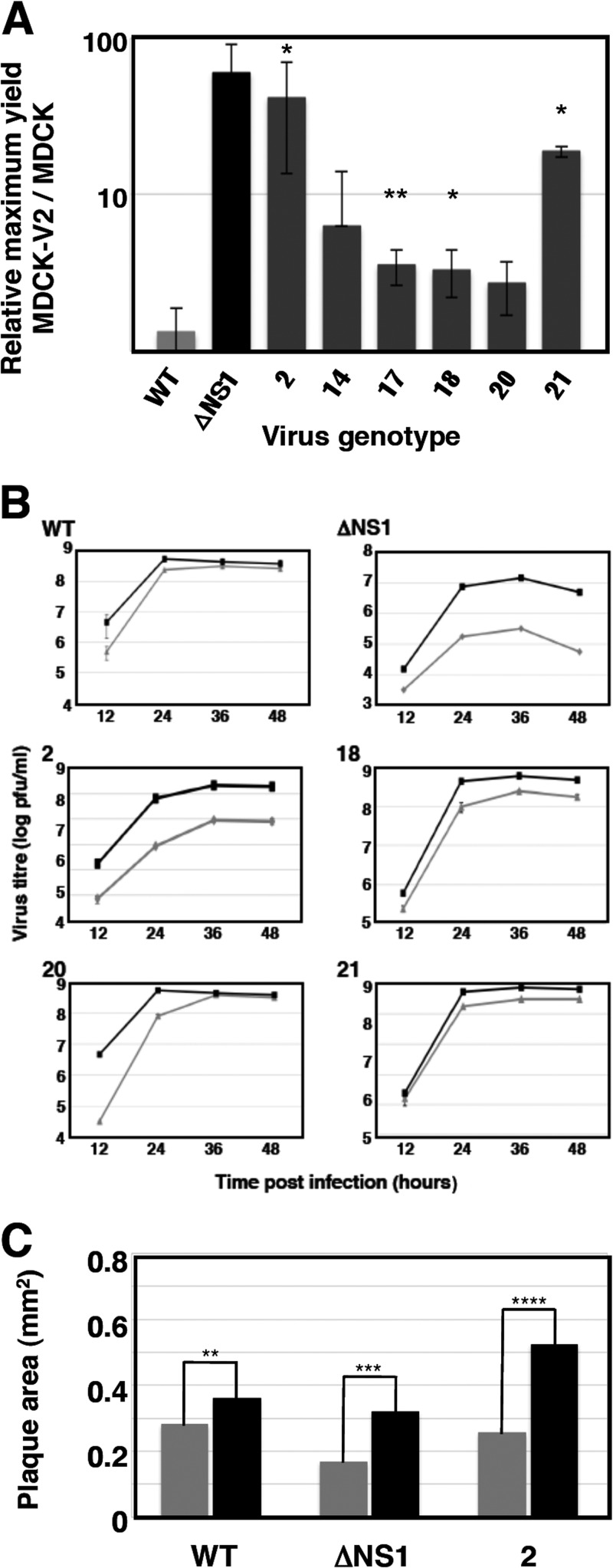
Replication efficiency of wt and individual mutant viruses in MDCK and MDCK-V2 cells. (A) The efficiency of replication of wt and various mutant viruses was evaluated by determination of maximal titers after low-multiplicity infections in either MDCK-V2 and MDCK cells, as indicated in Fig. 2. Values are averages and standard deviations from 3 experiments. *, *P* < 0.05; **, *P* < 0.01. (B) Kinetics of virus multiplication at low MOI. The titers at various times postinfection of MDCK-V2 (black lines) or MDCK (gray lines) cells were determined by plaque assay in MDCK-V2 cells. (C) Plaque size analysis of wt or mutant viruses. Wt or mutant viruses (mutant 2 or ΔNS1) were used for plaque assay in parallel in MDCK-V2 or MDCK cell cultures. After staining, the plaque size was determined using Image J software. The numbers of plaques measured were 176 (wt), 229 (ΔNS1), and 116 (mutant 2) in each cell line. **, *P* < 0.01; ***, *P* < 0.001; ****, *P* < 0.0001.

To isolate individual virus mutants potentially affected in their activation of the IFN response, single cells scored as positive for GFP expression after infection of A549/pr(IFN-β).GFP cells with the passage 10 V1 to V6 viruses at a low MOI (0.04 PFU/cell) were plated individually onto microcultures of MDCK-V2 cells, and the supernatants of cells showing a cytopathic effect were collected. Out of around 1,200 wells containing individual GFP-positive cells, viruses were recovered from 109 wells after endpoint dilution. There are a variety of possible reasons why not all GFP-expressing cells yield infectious progeny. For example, it has been shown that every virus population contains many mutants that are not viable but can express virus proteins or even replicate intracellularly (67); alternatively, some infected cells may be coinfected with defective particles that interfere with the replication of wt viruses. Around 75% of these viable viruses showed GFP expression higher than that of wt virus when tested by reinfection of A549/pr(IFN-β).GFP cells, and 6 of these were chosen for further analysis after preliminary phenotype characterization. First, the relative virus yield in IFN-responsive versus non-IFN-responsive cells was measured (Fig. 3). All selected mutants replicated efficiently in multicycle experiments in non-IFN-responsive cells. In comparison, in IFN-responsive cells, mutant 2 grew to much lower titers (Fig. 3A), while mutants 18, 20, and 21 yielded smaller reductions or slower kinetics (Fig. 3B). Mutants 14 and 17 gave similar final virus yields in both cell types, although mutant 14 showed protracted kinetics in IFN-competent cells (data not shown). Comparison of virus plaque size in MDCK versus MDCK-V2 cells revealed that only mutant 2 showed a reduction in plaque size in IFN-responsive cells, which was comparable to that of ΔNS1 virus (Fig. 3C). Furthermore, analyses of virus protein expression and localization during high-multiplicity infections in A549 cells showed only minor differences from wt virus (data not shown).

**FIG 4:**
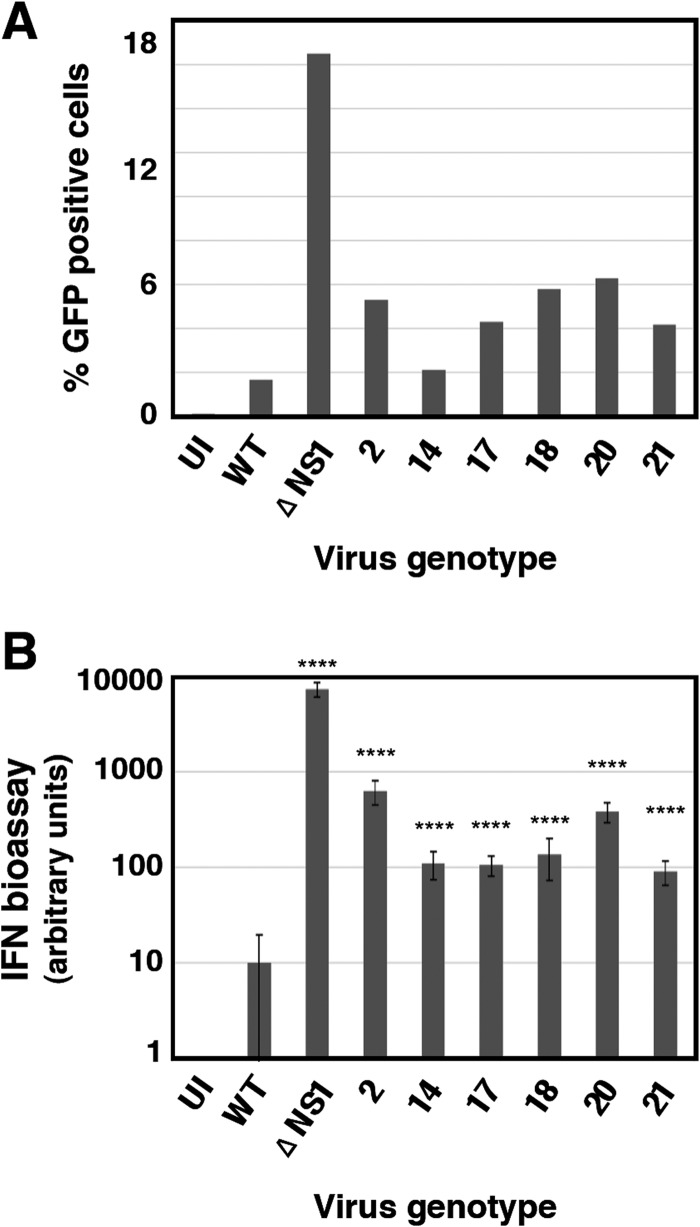
Induction of interferon by wt or mutant influenza viruses. (A) Virus progeny from individual A549/pr(IFN-β).GFP cells that were sorted as GFP positive and infected at low multiplicity was recovered after limiting-dilution infections in MDCK-V2 cells. The ability of these viruses to induce GFP expression after infection of A549/pr(IFN-β).GFP cells was quantified by FACS, using wt and ΔNS1 viruses as references. The results are representative of 3 independent experiments, in which 10,000 events were measured for each sample. (B) The ability of mutant viruses to induce the secretion of antiviral factors was tested by infecting of A549 cells at 5 PFU/cell, using wt and ΔNS1 viruses as references. Culture supernatants were collected at 24 hpi, and their ability to interfere with EMCV infection of A549/BVDV-Npro cells was determined by endpoint dilution, as described in Materials and Methods. ****, *P* < 0.0001.

**FIG 5:**
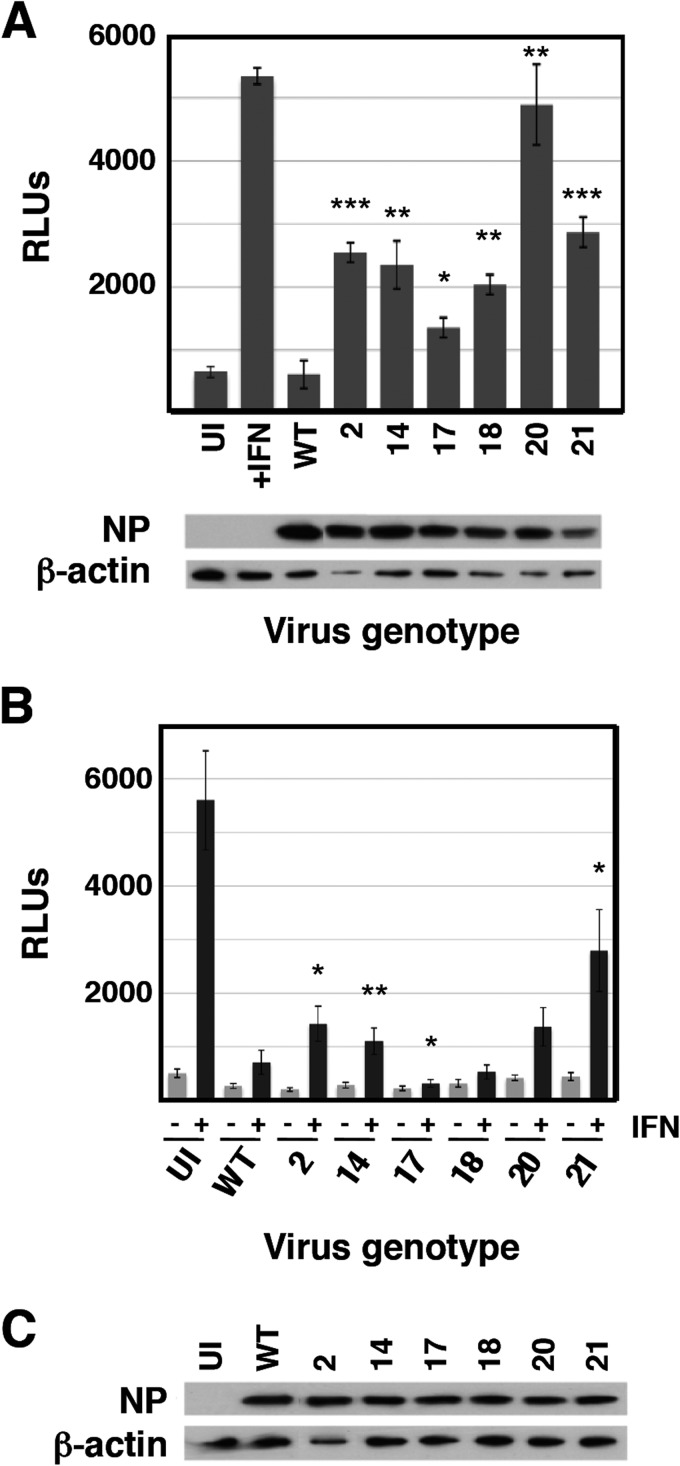
Activation of the ISRE by wt and mutant viruses. Cultures of A549/pr(ISRE).Luc (A) or A549/pr(ISRE).Luc-Npro (B) cells were infected at an MOI of 5 PFU/cell or mock infected with the indicated mutants or wt virus as a reference. A549 NPro/ISRE-luc cells constitutively express the BVDV NPro protein, which degrades IRF3; as a result, these cells cannot produce endogenous IFN (54). At 7 hpi, IFN was added to the indicated samples, and at 13 hpi, total cell extracts were prepared and luciferase activity was determined as described in Materials and Methods. Virus infection was verified by determination of NP accumulation by Western blotting, using β-actin as a loading control (C). *, *P* < 0.05; **, *P* < 0.01; ***, *P* < 0.001.

Next we analyzed the interplay of these mutants with the infected cell. The expression of GFP after high-multiplicity infection of A549/pr(IFN-β).GFP cells with the 6 selected mutant viruses is shown in Fig. 4A. Consistent with these results, all mutant viruses triggered the expression of antiviral factors to levels 10- to 50-fold higher than wt virus after high-multiplicity infection of human epithelial A549 cells, although this was lower than the induction observed following a parallel infection with ΔNS1 virus (Fig. 4B). Likewise, all mutant viruses were able to induce the expression of IFN-inducible genes, as demonstrated by the accumulation of luciferase reporter upon high-multiplicity infection of A549 ISRE-luc cells, in contrast to wt virus infection, which does not activate luciferase expression (Fig. 5A). In addition, some of the mutant viruses (mutants 2, 14, and 21) had a reduced ability, compared to wt virus, to block the upregulation of luciferase by exogenously added IFN (Fig. 5B).

**FIG 6:**
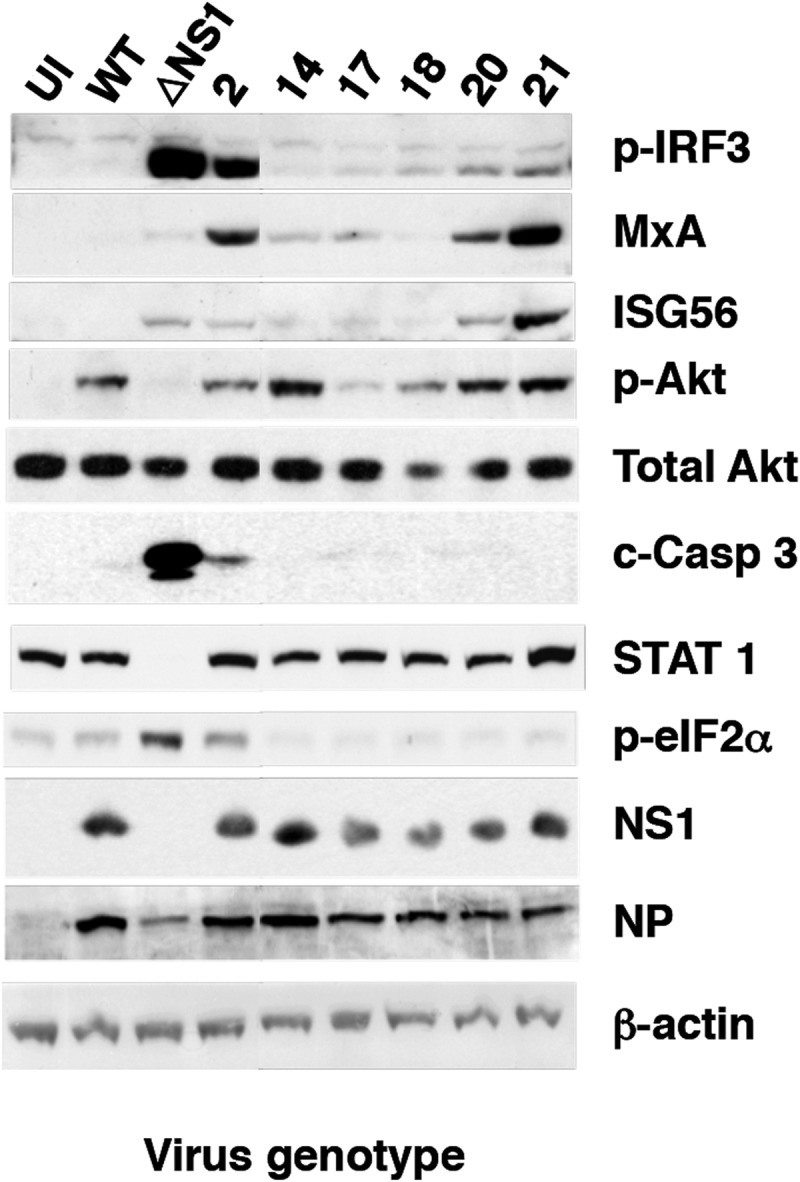
Activation of the interferon response by wt or mutant influenza virus infection. The induction of ISGs (MxA and ISG56) and activation of cell signaling pathways (p-IRF3, p-Akt, c-Casp3, STAT1, and p-eIF2a) were analyzed by Western blotting with specific antibodies at 16 hpi with the indicated mutant viruses, using uninfected cells (UI) and cells infected with wt or ΔNS1 viruses as references. Virus infection was detected by Western blotting with antibodies specific for NS1 and NP, using β-actin as a loading control.

To further analyze the interplay of these mutant viruses with the host cell, the expression of ISGs and the induction of specific signaling pathways were determined by Western blotting following high-multiplicity infection, using wt and ΔNS1 viruses as references (Fig. 6). These studies revealed the grouping of the mutant viruses into three phenotypes. Infection with mutant 2 led to a response somewhat similar to that observed in ΔNS1 virus infection: strong activation of IRF3, phosphorylation of eIF2α (a marker of PKR activity), and partial induction of apoptosis. In addition, mutant 2 infection showed induction of MxA, which was not observed for ΔNS1 virus infection (Fig. 6), probably as a consequence of ΔNS1 inducing apoptosis, leading to subsequent degradation of STAT1 by caspases (68). Mutants 20 and 21 behaved similarly; their infection led to partial activation of IRF3, expression of ISGs (MxA and ISG56), and activation of Akt (a consequence of NS1-dependent activation of PI3K [69]) (Fig. 6), but neither of these mutants induced apoptosis significantly. Of note, no strict correlation was observed between the levels of expression of ISGs and the corresponding levels of IFN activity secreted (Fig. 4B). These differences may be a consequence of the different kinetics of activation of these proteins. Finally, mutants 14, 17, and 18 did not show a clear phenotype in these assays, other than robust activation of Akt by infection with mutant 14.

### Genotype of individual virus mutants with an altered response to interferon.

To identify the genetic alterations present in the mutants described above that could be responsible for their observed phenotypes, their full genome sequence was determined by deep sequencing of virion RNA. The results are presented in Table 2. Nucleotide changes observed in the consensus sequence of the mutants compared with the initial virus used to start serial passages are in red. In addition, nucleotide changes that, although not altering the consensus sequence, reflect a significant proportion of the virus sequences at the corresponding position (i.e., higher than 30%) are in blue. The proportion of the alternative nucleotide in all positions recorded (percent alternative nucleotides) and the genomic heterogeneity found in the original virus stock (percent variation in the initial, nonpassaged virus [Mi]) and in the virus isolated after sorting of cells infected with the control virus populations (percent variation in MS) are also presented as a reference. Some of the recorded positions were very variable in the control virus populations and are highlighted in yellow, as for example mutant 2, position HA 700. We infer that these positions in the virus sequence can easily drift and therefore consider them probably irrelevant for the phenotype of the corresponding mutant. Other positions have very low variability in the control virus populations (see for instance mutant 2, position NS 217); these are highlighted in orange and were considered potentially relevant for the virus phenotype. A number of these nucleotide changes led to mutations at the protein level, and the amino acid changes are indicated (Table 2). Some corresponded to positions that had low variation in the control viruses and that were phylogenetically conserved in the influenza A database (highlighted in blue) or showed low phylogenetic variation (highlighted in light blue). Sequence analysis of virus populations passaged in non-IFN-responsive cells and selected by cell sorting showed that most of the amino acid changes detected did not map to the NS1 protein. In fact, the only NS1 mutation observed was I64T (NS 217) in the NS1 of mutant 2; none of the other mutant viruses encoded altered NS1 proteins. The phenotype of mutant 2 was similar to that of ΔNS1 virus, for which it is tempting to speculate that mutation NS1 I64T is responsible. The relevance of the mutations identified in mutants 14 to 21 is not clear at this point, although it is noteworthy that mutants 17 to 21 contain alterations in either M1 (mutant 18, I115M, and mutants 17, 20, and 21, D30N) or M2 (mutants 17, 20, and 21, A86S). The mutations M1-I115M and M2-A86S have been detected very seldom (either 0/6,607 or 1/6,456-fold) (Table 2) and could be relevant for the mutant phenotype. Mutant 14 has a mutation in the polymerase domain of PB1 (N455D) (Table 2) that could alter its RNA replication efficiency and be the reason for its protracted replication kinetics.

**TABLE 2:**
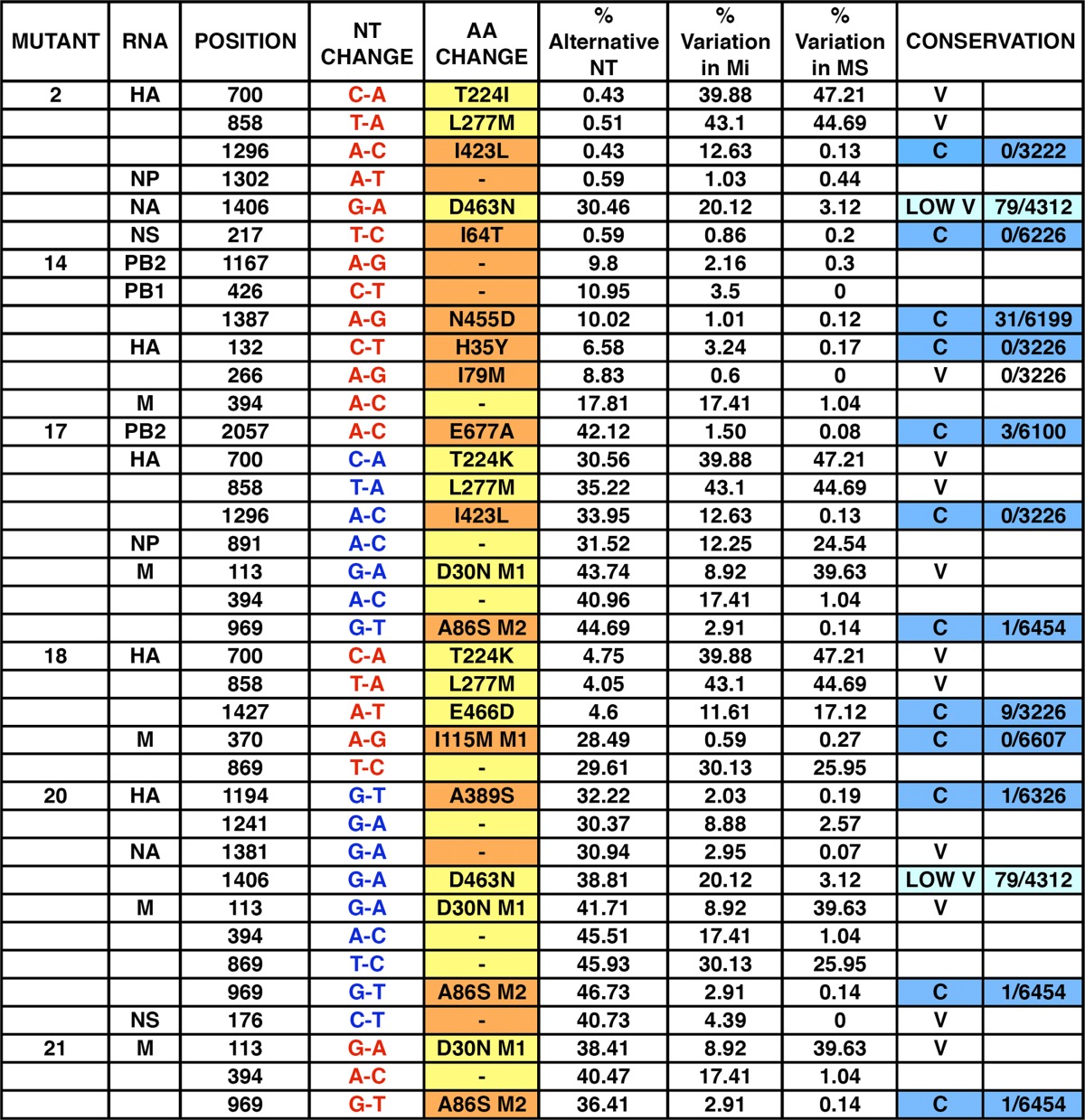
Mutations detected in individual virus mutants^*a*^

a The mutations detected in individual virus mutants are presented. Mutations that appear as such in the consensus sequence are in red. Those that do not appear as mutations in the consensus sequence but are present in a proportion over 30% of the virus sample are indicated in blue. Amino acid changes in yellow are very variable in the control virus populations, and those in orange have very low variability in the control virus populations. For comparison, the frequencies of these mutations in the initial, nonpassaged virus (Mi) and in a control population passaged in MDCK cells and sorted for GFP expression (MS) are also presented. The conservation of these positions in the influenza virus sequence database is denoted as conserved (C; blue), low variable (LOW V; light blue; 10 < *n* < 100), or variable (V; *n* > 100), and the number of instances each mutation appears among the total number of sequences screened is indicated.

**FIG 7:**
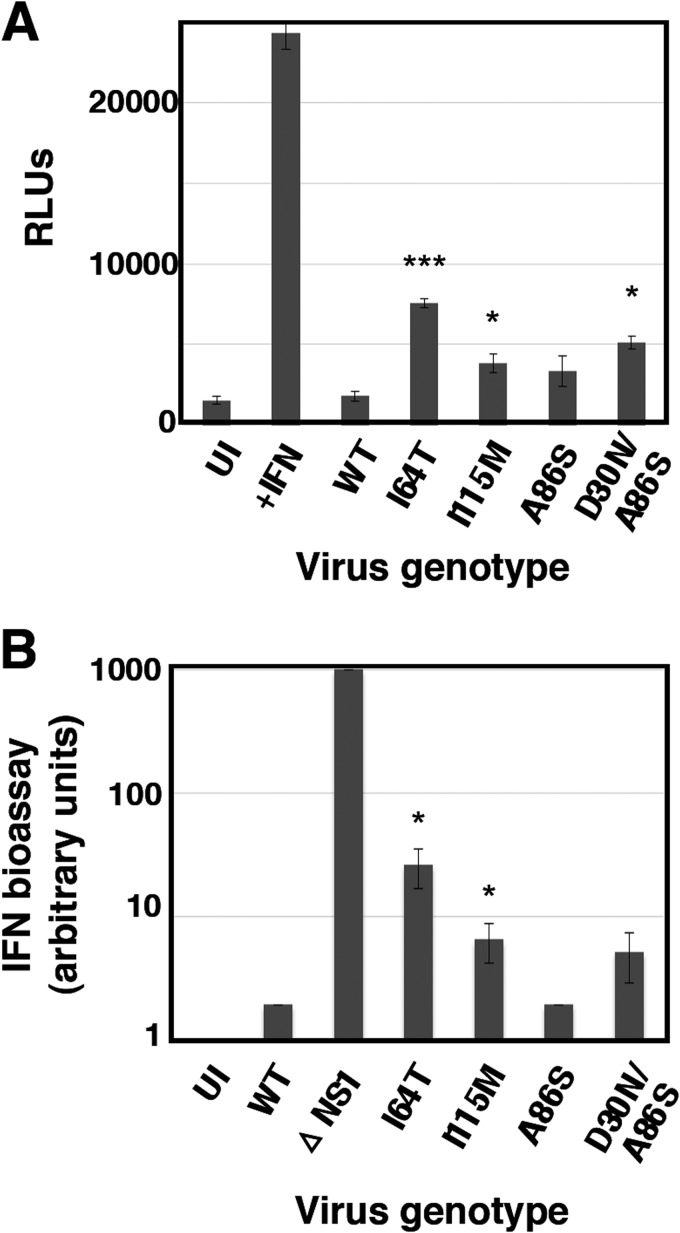
Phenotype of recombinant virus mutants. The capacity of mutant viruses to induce luciferase under the control of an ISG promoter (A) was tested by high-multiplicity infection of A549/pr(ISRE).Luc, using wt virus as reference. The capacity of mutant viruses to induce the secretion of antiviral factors (B) was tested in infections of A549 cells at 5 PFU/cell, using wt and ΔNS1 viruses as references. Culture supernatants were collected at 24 hpi and their ability to interfere with EMCV infection of A549/BVDV-Npro cells was determined by endpoint dilution and is presented in arbitrary units. *, *P* < 0.05; ***, *P* < 0.001.

To verify whether the mutations identified in this screen were relevant for the observed phenotype, some of them were introduced into infectious virus by reverse genetics using the VIC virus genetic background. Specifically, rescue of mutations NS1-I164T, M2-A86S, M1-I115M, and M1-D30N was attempted. All rescued viruses were viable except mutant M1-D30N, which could be rescued only in combination with mutation M2-A86S (i.e., as found in mutants 17, 20, and 21, described above). To analyze the phenotypes of these recombinant viruses, the abilities of rescued viruses to activate expression of an ISRE-responsive luciferase reporter and to induce IFN secretion from infected cells were examined (Fig. 7). Recombinant mutants NS1-I64T, M1-I115M, and M1-D30N/M2-A86S showed luciferase inductions similar to those observed in the original mutants 2, 20, and 21, respectively, while mutation M2-A86S alone did not show a clear phenotype. Similar results were obtained for IFN induction (Fig. 7B).

### Some IFN-inducing virus mutants are particularly prone to generating defective interfering particles.

**FIG 8:**
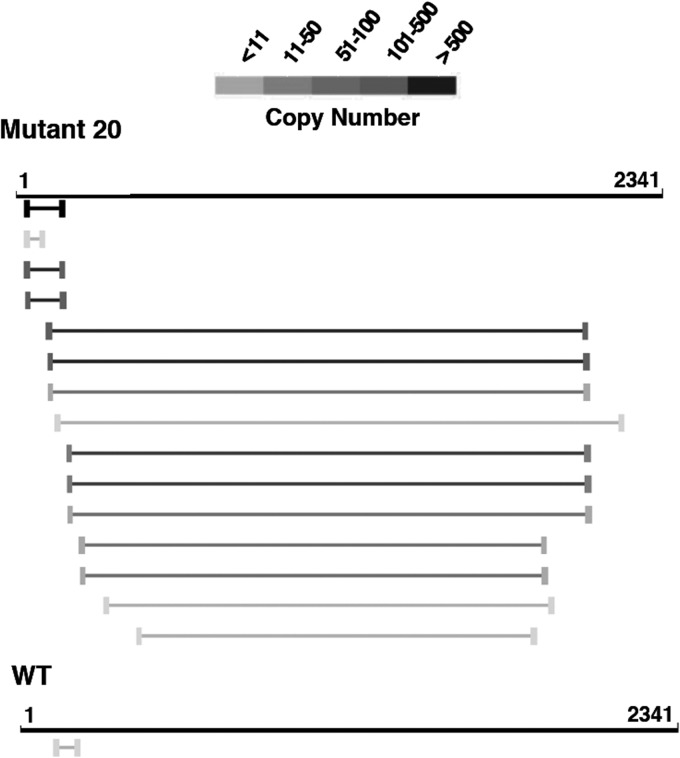
Deletion-containing virus RNAs detected by deep sequencing. The figure shows an example of the results obtained by bioinformatics analysis of the deep-sequencing data of wt and mutant virion RNAs (PB1 RNA segment; comparison of mutant 20 and wt virus). The bars indicate the sequence deletions mapped in the RNA segment coordinates. The gray scale indicates the frequency of each particular deletion in the deep-sequencing data. Only deletions larger than 15 nt and present more than 5 times are shown. The complete deletion data set for all viruses is available via the supplemental material.

**FIG 9:**
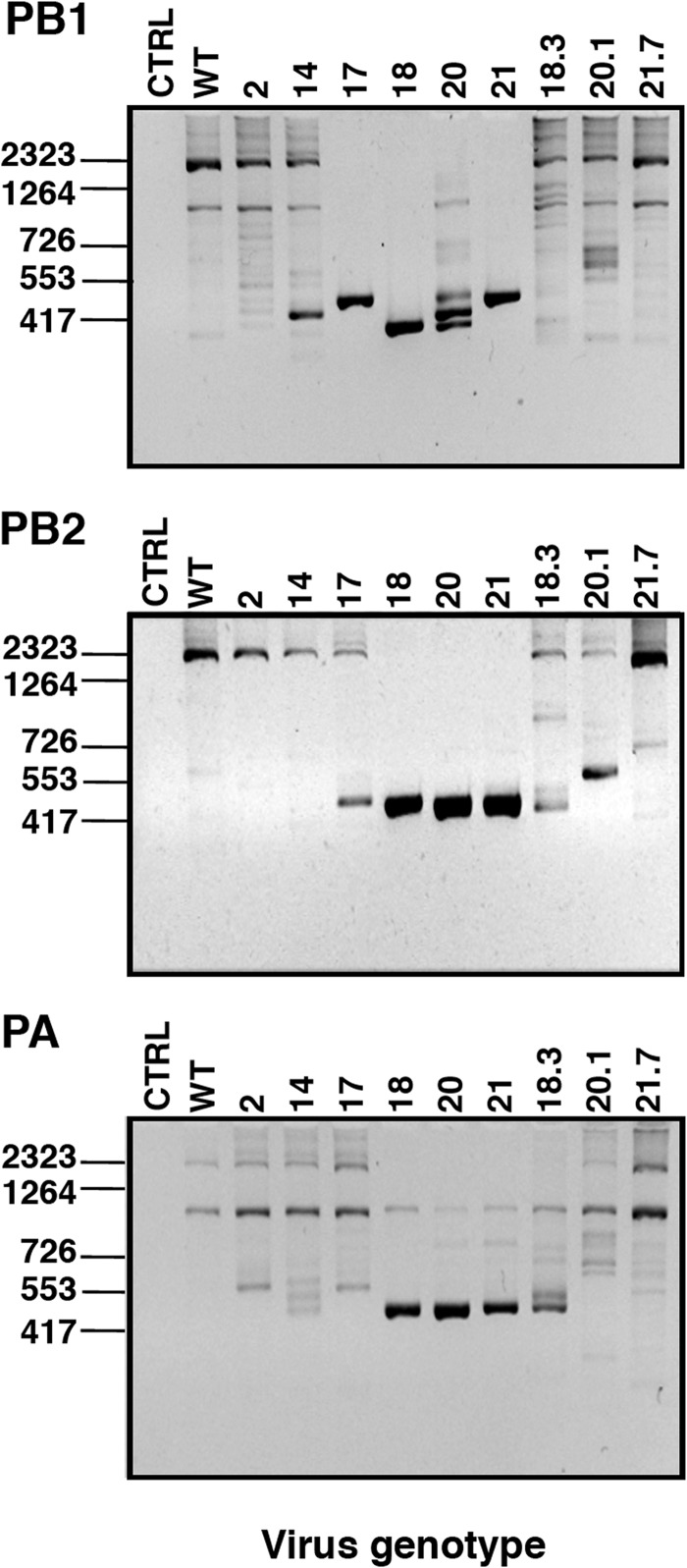
Experimental verification of the presence of deleted genomic RNAs. RNA samples from purified wt or mutant 2, 14, 17, 18, 20, and 21 virions were used for RT-PCR using terminal oligonucleotide primers corresponding to the PB1, PB2, and PA RNA segments, and the PCR products were analyzed by agarose gel electrophoresis. For mutants 18, 20, and 21, individual plaques were isolated (plaques 18.3, 20.1, and 21.7), amplified, and used for RT-PCR and agarose gel electrophoresis as described above.

Since the induction of IFN has been associated with the presence of defective interfering (DI) viruses in both influenza viruses and paramyxoviruses (70–73), we used bioinformatic analysis of the deep-sequencing data for each mutant virus to detect and quantitate the presence of internal deletion DI RNAs in the purified virions. An example of the results is presented in Fig. 8, the summary of deletion-containing RNA accumulations is shown in Tables 3 and 4 and the complete data set is available via the supplemental material. A large number of deletions were observed that were individually very infrequent and widely distributed across all genes of the various viruses (although they are clearly more abundant in the polymerase genes) (Table 3). These deletions could be interpreted as the result of common polymerase errors during replication. However, they would not be efficiently amplified and/or encapsidated, and therefore each one was identified only a few times in the data set. More abundant deletions were identified that were preferentially derived from a subset of viral genes and were differentially represented in the wt and mutant viruses (Table 4). Among the abundant deletions, two size classes could be distinguished: short (100 to 200 nt) (Table 4; also, see Fig. 8 as an example) or long (almost 2,000 nt) (Table 4; also, see Fig. 8 as an example). The abundant RNAs containing short deletions were detected exclusively from the PB2 segment of mutants 18 and 20 and were not detectable in other segments or mutants (Table 4); the origin of these differences is unclear at present. The abundant RNAs containing long deletions were exclusively derived from the polymerase genes (Table 4 and Fig. 8; also, see the supplemental material) and could represent classical DI RNAs (74). The accumulation of long-deletion RNAs was many-fold higher in mutants 18, 20, and 21 than in the other mutants or the wt virus, as determined by analysis of the deep-sequencing data (Table 4; also, see the supplemental material), with mutant 17 representing an intermediate situation. The accumulation of deletion-containing RNAs by specific mutants was verified by PCR amplification using terminal oligonucleotide primers specific for the polymerase genes (Fig. 9). Although preparations of all mutants were prepared in parallel at low MOI, it could be argued that these disparities arose from different passage histories of the various mutants. To rule out such a possibility, mutants 18, 19, and 20 were plated on MDCK-V2 cells, individual plaques were picked, and the viruses were tested for the presence of deletion-containing RNAs as described above. The results indicated that these mutants generated deletion-containing RNAs very frequently (Fig. 9, lanes 18.3, 20.1, and 21.7). Interestingly, the sizes of the DI RNAs observed after plaque purification were different from those that accumulated in the corresponding (non-plaque-purified) mutant virus stock, indicating that they arose as new deletions in the single plaques and were not carried over from the original virus.

**TABLE 3:** Total accumulation of deletion-containing RNAs in wt and mutant viruses

Virus	No. of total deletion-containing RNAs in segment^*a*^															
	PB2		PB1		PA		HA		NP		NA		M		NS	
	L	S	L	S	L	S	L	S	L	S	L	S	L	S	L	S
Wt		2		5		2		2		27		8	4*	4	24*	6
2	7	6	29	3	35	5							5*		35*	
14	4	24	44	20	150	6				2				6	5*	
17	7	12	549	6	115	6		2		7		9		2	6*	
18	756	37	120	254	831	16				2	2	4		21	2*+7	4
20	2,136	98	848	2,698	3,434	72	18	24	20	10	20	30	*6 + 8	34	14*+4	18
21	676	15	564	18	1,101	18				2			2*		5*	

a The number of individual deletion-containing RNAs showing deletions longer than 15 nt that appear at least twice in each sample is given. Data were standardized according to the number of deep-sequencing reads obtained for each virus sample and are presented as numbers of short deletions (S; 100 to 200 nt) or long deletions (L; around 2,000 nts). Asterisks denote spliced mRNAs from segments M and NS that contaminated the virion preparation.

**TABLE 4:** Accumulation of abundant deletion-containing RNAs in wt and mutant viruses

Virus	No. of abundant deletion-containing RNAs in segment^*a*^															
	PB2		PB1		PA		HA		NP		NA		M		NS	
	L	S	L	S	L	S	L	S	L	S	L	S	L	S	L	S
Wt																
2															20*	
14					113											
17			456													
18	756		59	248	756											
20	2,096		728	2,632	3,294											
21	632		472		1,005											

a The number of individual deletion-containing RNAs showing deletions longer than 15 nt that appear at least 20 times in each sample is given. Data were standardized according to the number of deep-sequencing reads obtained for each virus sample and are presented as numbers of short deletions (S; 100 to 200 nt) or long deletions (L; around 2,000 nt). The asterisk denotes a spliced mRNA from segment NS that contaminated the virion preparation.

## DISCUSSION

### Complex interaction between influenza virus and the cellular innate immune response.

**FIG 10:**
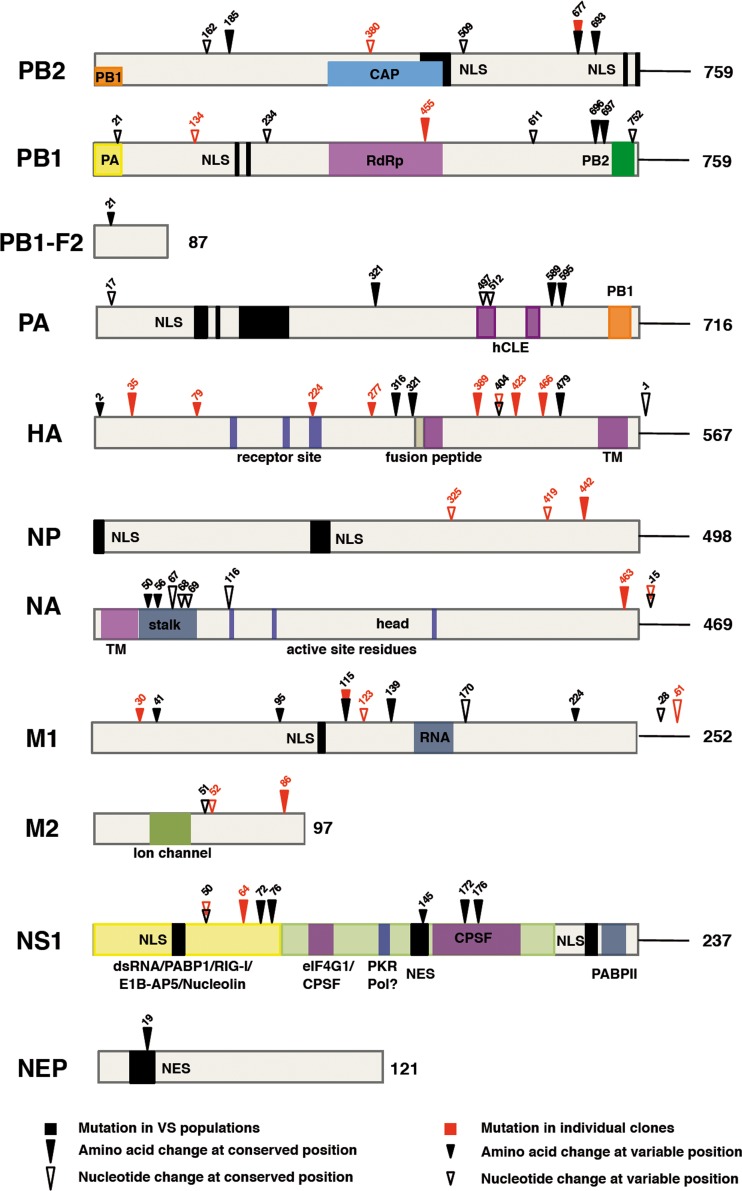
Mapping the mutations observed in selected virus populations and mutants. The diagram shows the location of mutations detected in virus populations and individual virus mutants derived by serial passage in non-IFN-responsive cells and sorting for IFN-dependent GFP expression. The bars show each virus-specific protein, and the numbers on the right denote the number of amino acids. Specific features of each protein are indicated. Arrowheads show the position of mutations observed in VS virus populations (black) or individual virus mutants (red). Filled arrowheads denote nonsynonymous mutations, whereas empty arrowheads indicate synonymous mutations. The arrowhead size indicates the phylogenetic conservation of the mutation site.

Like many other viruses, influenza A viruses need to counteract the cellular innate immune response in order to achieve a productive infection. Most of this viral counteraction is mediated by the NS1 protein (21, 32, 33, 75), although PB2 has also been implicated (44, 45). The approach taken here naïvely questioned the genetic solutions that the virus could adopt within its sequence space to optimize its replication under non-IFN-responsive conditions and hence could identify other constraints imposed by the innate immune system. The mutations identified in several virus populations that replicate to higher titers in non-IFN-responsive cells than in IFN-competent cells presumably include genetic solutions which are normally restricted in an IFN-responsive system but which viruses are allowed to adopt when the innate immune response is ablated. The experimental approach used does not necessarily guarantee that identified mutations in the selected virus populations affect positions normally involved in restricting the IFN response. However, two observations suggest that this is likely: (i) the repetitive identification of a fraction of these mutations in several virus populations that evolved independently and (ii) the observation that most of the amino acid changes detected affect very phylogenetically conserved positions. In addition, a number of mutations were identified in individual IFN hyperinducer virus clones selected in parallel. The complete set of mutations is summarized in Fig. 10. It is clear that amino acid changes were not restricted to the NS1 protein. Rather, the HA, M1, and polymerase genes showed a number of mutations comparable to that observed in NS1, while NP and NA were the least mutated genes. Among the amino acid changes detected in the polymerase, PB2-G693E stands out as potentially altering the nuclear localization signal (NLS) and hence importin-alpha binding (76), while PA-L589I is located at the PB1 binding site (77, 78). Two amino acid changes were observed at positions phylogenetically conserved within the NS1 protein effector domain; E172K and N176I localize to the cleavage and polyadenylation specificity factor (CPSF)-binding region (35, 79–82). Finally, M1-T139I affects a conserved position in the protein sequence that is located at the dimer interaction surface in the M1 crystal structure (83, 84). However, this genomic analysis cannot discern whether these mutations affect directly virus counteraction of IFN, since they could also reflect random passenger or compensatory mutations that might be linked to the relevant ones. Altogether, the landscape of mutations suggests that the virus has found multiple solutions in its adaptation to efficiently replicate in a non-IFN-responsive system, and among the mutants selected by IFN-dependent GFP expression, only a fraction had mutations that affect the well-known NS1 modulator of the innate immune response. These results are in contrast with a recently published mutagenesis study of the influenza genome (85) in which only the hemagglutinin head and NS1 protein showed sufficient structural flexibility to allow small insertions without altering virus viability. Altogether, the results presented here indicate that under normal replication conditions, the virus sequence space is restricted in essentially every gene by the cellular innate immune response and ablating the IFN response allows the virus to explore new possibilities compatible with good replication fitness, some of which affect levels of IFN induction.

### Poor IFN blockers or IFN hyperinducers?

The phenotypic and genotypic analyses of the individual virus clones selected showed two distinct groups, one represented by clone 2 and the other including clones 18, 20, and 21 and to a lesser extent clone 17. Clone 2 virus is phenotypically similar to ΔNS1 virus, as it cannot restrict IFN induction or IFN-induced ISG expression and contains an I64T mutation in NS1. This suggested that this single change is sufficient to abolish the virus capacity to counteract the IFN-mediated response to infection, a contention that was verified by the phenotype of the recombinant virus containing NS1-I64T as a single mutation (Fig. 7). In contrast, the clone 18/20/21 group of viruses did not show mutations in NS1 protein, and hence their capacity to counteract the IFN response should be normal. However, these mutants still induced higher levels of IFN than wt virus. This apparent contradiction could be explained by the fast generation of deleted genomic RNAs in the 18/20/21 group compared to wt or clone 2 viruses. Whereas all viral clones tested generated a low abundance of deletions essentially affecting many viral RNAs, the 18/20/21 group of viruses accumulated large numbers of deletion-containing RNAs originating only from the polymerase segments, in line with the early studies characterizing influenza DI RNAs (74, 86). However, the clone 18/20/21 group of viruses were unable to block IFN-induced ISG expression, and the mechanism underlying this defect is still unclear, but it may be a result of DI RNA-mediated interference with nondefective virus replication. Surprisingly, viral clones 18, 20, and 21 showed mutations not in the polymerase genes but in the M1/M2 proteins, and the relevance of mutations M1-I115M and M1-D30N was verified by the phenotype of the corresponding recombinant viruses (Fig. 7). One possible explanation is that these mutants are particularly prone to the encapsidation of deletion-containing RNAs into the progeny virions (11, 87). Surprisingly, this does not lead to low virus titers (Fig. 3 and data not shown), suggesting that deletion-containing RNAs are incorporated in addition to normal-segment RNAs. As RIG-I has been shown to preferentially associate with short influenza virus RNAs, including DI RNAs from the polymerase segments (70), the properties of 18/20/21 viruses would suggest that they are particularly good inducers of the IFN response due to increased accumulation of deletion-containing RNAs, rather than poor blockers of IFN induction (since they possess intact NS1 proteins), in line with the recent report showing the importance of DI RNAs in the innate response *in vivo* (88). This phenotype, together with the surprisingly high fitness of these viruses, suggests their potential use as attenuated strains.

In summary, by serial passage of influenza viruses in non-IFN-responsive cells, we have obtained virus populations and individual virus clones that replicate better in these cells than in IFN-responsive cells and are better able to induce IFN and IFN-inducible genes than wt virus. The landscape of mutations identified indicates that most of the mutant viruses express a wt NS1 protein and that the mutations are widely distributed among all viral genes. This suggests that essentially the entire virus gene complement is tuned to counteract the IFN response and that it drifts away from such an optimal sequence when the IFN response is ablated.

## Supplementary Material

Supplemental material
