# Supplementary material for: An Unbiased Genetic Screen Reveals the Polygenic Nature of the Influenza Virus Anti-Interferon Response
Source: J Virol. 2014 May;88(9):4632–46. doi: 10.1128/JVI.00014-14 (PMC3993829; doi:10.1128/JVI.00014-14)
Supplement: Supplemental material [file supp_88_9_4632__index.html]

An Unbiased Genetic Screen Reveals the Polygenic Nature of the Influenza Virus Anti-Interferon Response — Supplemental material 

# An Unbiased Genetic Screen Reveals the Polygenic Nature of the Influenza Virus Anti-Interferon Response

## Supplemental material

**Files in this Data Supplement:**

- Supplemental file 1 -

  Supplemental Information S1 (Link to complete data set of deleted virus RNAs.)

  PDF, 38K
