# Supplementary material for: An Unbiased Genetic Screen Reveals the Polygenic Nature of the Influenza Virus Anti-Interferon Response
Source: J Virol. 2014 May;88(9):4632–46. doi: 10.1128/JVI.00014-14 (PMC3993829; doi:10.1128/JVI.00014-14)
Supplement: Supplemental material [file JVI.00014-14_zjv999098949so1.pdf]

Supplementary information. The following link can be used to inspect the complete dataset of deleted virus RNAs:

http://bioinfogp.cnb.csic.es/public/deletions_viewer
